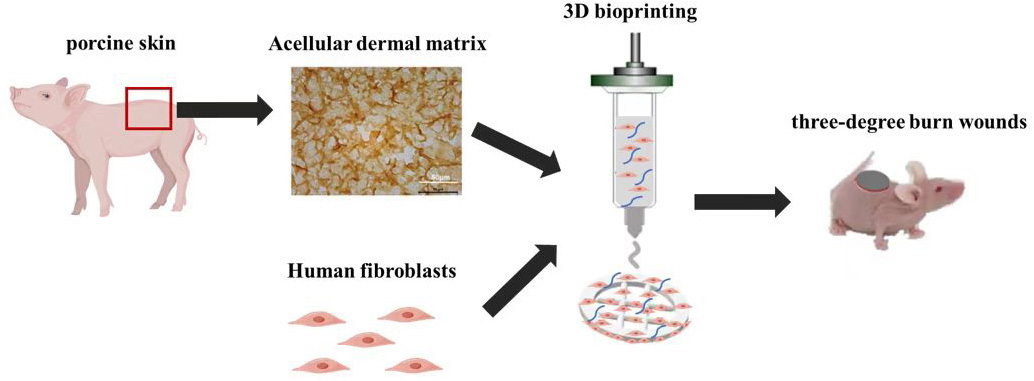# 820 Photocrosslinkable Acellular Dermal Matrix Bioink Promotes Beneficial Cellular Behavior and Wound Healing

**DOI:** 10.1093/jbcr/iraf019.351

**Published:** 2025-04-01

**Authors:** Ronghua Jin

**Affiliations:** The Second Affiliated Hospital of Zhejiang University College of Medicine

## Abstract

**Introduction:**

Bioinks are highly important for three-dimensional (3D) bioprinting. Acellular dermal matrix (ADM), a decellularized matrix derived from the dermis, has attracted attention as a potential bioink because it provides a microenvironment similar to that of living cells. However, its poor printability and weak mechanical properties make it difficult to apply widely in 3D bioprinting.

**Methods:**

For swelling ratio measurements, six sample from each group were immersed into PBS at 37°C for 24 h and measured the weights of the hydrated gels (Wwet). Then, the hydrated gels were freeze-dried for 24 h and measured the weight (Wdry). The swelling ratio was calculated according to the equation: SR = (Wwet - Wdry)/ Wdry × 100%.Rheological analysis was performed by using a rotational rheometer.Young’s modulus was measured by using a Park XE-70 atomic force microscopy and Triangular silicon nitride cantilever. Cell viability was assessed by a live/dead cell stain according to the manufacturer’s protocol after 1 and 7 days of cultivation in vitro. Cell proliferation was determined by the Cell Counting Kit-8 (Boster, China) according to the manufacturer’s protocol after 1 and 7 days of cultivation in vitro. In vivo, Mice were divided into three groups: blank, A+G and A+G+cell, which respectively treated with nothing, ADM-GelMA bioink and bioprinted ADM-GelMA fibroblast-laden construct. The bioprinted constructs with skin and surrounding tissues were sampled for histological analysis on Day 14 and 21.

**Results:**

1.Characterization of ADM:no nuclei stained blue with DAPI were observed in the ADM sections. IHC and IF staining were used for qualitative evaluation of the key ECM components after decellularization, and collagen I and collagen III were well preserved.2.Swelling ratio, rheology and printability of the ADM-GelMA bioink: The swelling ratio of the ADM-GelMA bioinks (22.27 ± 3.77) was lower than that of the ADM bioinks (42.26 ± 1.09) but greater than that of the GelMA bioinks (11.02 ± 0.89).The shear-thinning properties of the ADM-GelMA and GelMA bioinks were more pronounced than those of the ADM bioinks.ADM-GelMA and GelMA bioinks exhibited relatively high fidelity, while ADM bioinks collapsed without gaps. 3.Cytocompatibility of ADM-GelMA bioinks:the cell proliferation in the ADM-GelMA and ADM bioinks was significantly greater than that in the GelMA bioink. 4. in vivo: the wound closure of the A+G+cell group was the fastest, and complete healing was achieved on D21.

**Conclusions:**

In this study, we developed a new bioink, a mixture of ADM and GelMA bioink, to increase the mechanical strength and printability of ADM while maintaining high cytocompatibility. Then, the ADM-GelMA construct was implanted in vivo to evaluate its ability to promote ECM formation and accelerate wound healing.

**Applicability of Research to Practice:**

we developed a new bioink for wound healing

**Funding for the Study:**

The National Natural Science Foundation of China (82202443)